# Interplay of Carbonic Anhydrase IX With Amino Acid and Acid/Base Transporters in the Hypoxic Tumor Microenvironment

**DOI:** 10.3389/fcell.2020.602668

**Published:** 2020-11-09

**Authors:** Geetha Venkateswaran, Shoukat Dedhar

**Affiliations:** ^1^Department of Integrative Oncology, British Columbia Cancer Research Centre, Vancouver, BC, Canada; ^2^Interdisciplinary Oncology Program, The University of British Columbia, Vancouver, BC, Canada; ^3^Department of Biochemistry and Molecular Biology, The University of British Columbia, Vancouver, BC, Canada

**Keywords:** tumor microenvironment, hypoxia, carbonic anhydrase IX, amino acid transport, tumor metabolism

## Abstract

Solid tumors are challenged with a hypoxic and nutrient-deprived microenvironment. Hence, hypoxic tumor cells coordinatively increase the expression of nutrient transporters and pH regulators to adapt and meet their bioenergetic and biosynthetic demands. Carbonic Anhydrase IX (CAIX) is a membrane-bound enzyme that plays a vital role in pH regulation in the tumor microenvironment (TME). Numerous studies have established the importance of CAIX in mediating tumor progression and metastasis. To understand the mechanism of CAIX in mediating tumor progression, we performed an unbiased proteomic screen to identify the potential interactors of CAIX in the TME using the proximity-dependent biotin identification (BioID) technique. In this review, we focus on the interactors from this BioID screen that are crucial for nutrient and metabolite transport in the TME. We discuss the role of transport metabolon comprising CAIX and bicarbonate transporters in regulating intra- and extracellular pH of the tumor. We also discuss the role of amino acid transporters that are high confidence interactors of CAIX, in optimizing favorable metabolic state for tumor progression, and give our perspective on the coordinative interplay of CAIX with the amino acid transporters in the hypoxic TME.

## Introduction

Tumor cells metabolize nutrients in an anabolic or catabolic mode to maintain their biosynthetic and bioenergetic demands, respectively. In a catabolic pathway, nutrients are broken down to generate energy for maintaining cellular integrity. Whereas, in an anabolic pathway, they are utilized to build new macromolecules such as nucleotides and amino acids that support cell growth and proliferation. Tumor cells can alter their metabolism in favor of either of these pathways based on their requirements, which is called metabolic reprogramming/rewiring (Ward and Thompson, 2012). Besides, tumor cells can utilize a diverse array of nutrients such as glucose, glutamine (Gln), essential amino acids, and fatty acids, thus offering them metabolic flexibility (DeNicola and Cantley, 2015). Both, metabolic reprogramming and flexibility give tumor cells the plasticity to adapt to any metabolic shifts and survive. Several cell-intrinsic and extrinsic factors influence metabolic reprogramming and flexibility in tumor cells (DeNicola and Cantley, 2015). One of the most important extrinsic factors is oxygen availability in the tumor microenvironment (TME) (Nakazawa et al., 2016). Solid tumors are characterized by chaotic, immature vasculature that causes zones of varying oxygen tensions within the tumor. Depending on the proximity to blood vessels, tumors are comprised of poorly perfused, chronic hypoxic zones, and intermittently perfused, cycling hypoxic zones (Michiels et al., 2016). To survive the nutrient and oxygen deprivation caused by insufficient perfusion, tumor cells trigger hypoxia-inducible factor (HIF) signaling, which culminates in the stabilization and activation of the transcription factor, HIF1α or HIF2α, and alters the expression of several downstream targets to promote survival, tumor growth and progression (Xie and Simon, 2017). One of the HIF1α regulated proteins that play an important role in the hypoxic TME is the Carbonic Anhydrase IX (CAIX) (Wykoff et al., 2000; McDonald and Dedhar, 2014).

## Caix – Function and Role in Cancer

Carbonic anhydrase IX is a dimeric, membrane-bound metabolic enzyme that belongs to the carbonic anhydrase (CA) family (Alterio et al., 2012). It plays a crucial role in pH regulation through the reversible hydration of carbon dioxide into bicarbonate and proton. CAIX comprises of extracellular facing proteoglycan (PG) and catalytic (CA) domains, a transmembrane (TM) domain, and an intracytoplasmic (IC) domain (Opavský et al., 1996). The presence of the PG domain is a unique feature of CAIX and is absent in other isozymes of the CA family. The dimerization of CAIX is mediated by the formation of a disulfide bond between the Cys-41 residue located on the CA domain (Alterio et al., 2009). Although CAIX expression is primarily driven under hypoxia through the HIF1α stabilization, the presence of extracellular lactate (Panisova et al., 2017) and glutamate (Glu) (Briggs et al., 2016) have also been shown to stabilize HIF1α and promote CAIX expression under normoxia. CAIX is predominantly expressed in solid tumors, with restricted expression in normal tissues (McDonald et al., 2012; Mboge et al., 2018) and, its expression can be correlated with poor prognosis (Chia et al., 2001; Loncaster et al., 2001; Klatte et al., 2009; Korkeila et al., 2009; Ilie et al., 2010) and response to therapy in solid tumors (Koukourakis et al., 2001; Generali et al., 2006; Tan et al., 2009; McIntyre et al., 2012). The role of CAIX in various steps of tumor progression and metastasis is well established in the past decade. Targeting CAIX, both, by genetic depletion and using small molecule inhibitors, has elucidated the importance of CAIX in tumor growth *in vivo* (Lou et al., 2011). In addition to its role in tumor growth, CAIX plays a crucial role in metastasis (Lou et al., 2011; Gieling et al., 2012; Chafe et al., 2015). Before the cancer cells metastasize to a distant site, they establish a conducive microenvironment for their survival, called the pre-metastatic niche. CAIX promotes granulocyte colony-stimulating factor (G-CSF) production by hypoxic breast cancer cells, which helps in the mobilization of granulocytic myeloid-derived suppressor cells to lung metastatic niche and primes for metastasis (Chafe et al., 2015). Furthermore, CAIX helps in the maintenance of stemness in cancer stem cells and favor metastasis (Lock et al., 2013; Gibadulinova et al., 2020; Peppicelli et al., 2020). While it is evident that CAIX is important in mediating various steps in tumor progression, the underlying mechanisms remain unclear. Considering the importance of CAIX in the hypoxic microenvironment, it is plausible that CAIX interacts with other proteins in tumor cells to mediate various functions. Hence, we recently conducted a comprehensive, unbiased study to identify the protein interactome of CAIX using the proximity-dependent biotinylation labeling technique called the BioID method (Roux et al., 2012). This study identified over 140 high confidence protein interactors of CAIX (Swayampakula et al., 2017). In this mini review, we will focus on the amino acid transporters (AATs) and acid/base transporters that were identified as high confidence interactors.

## Caix and pH Regulation

Active metabolism within tumor cells leads to the accumulation of acidic metabolic by-products, which, if unbuffered, will be lethal to the tumor cells. Therefore, tumor cells deploy several membrane acid/base transporters (pH regulators) to establish a favorable pH within the tumor cells (Neri and Supuran, 2011). Two major acidic metabolic by-products that are produced by tumor cells are CO_2_ and lactic acid (Corbet and Feron, 2017). While CO_2_ is predominantly produced as a by-product of aerobic respiration, lactic acid production is a result of anaerobic respiration or aerobic glycolysis in tumor cells. The CO_2_ generated by tumor cells acts as a substrate for CAs, to produce bicarbonate and protons. Lactic acid, on the other hand, is extruded out of the cells by monocarboxylate transporters (MCT) as lactate and protons, or buffered intracellular by bicarbonate ions to produce CO_2_ (Sun et al., 2020). The contribution of CO_2_ and lactic acid in defining the intratumoral pH will depend on factors such as oxygenation and mitochondrial respiration in tumor cells. In deep hypoxic zones of a tumor, the mitochondrial respiration is impeded, and therefore, glycolysis becomes the primary state of metabolism (Corbet and Feron, 2017). Conversely, in moderately hypoxic zones of the tumor, the lactate that is released by surrounding anaerobic cells or Gln imported into cells can feed the TCA cycle and drive oxidative phosphorylation (Corbet and Feron, 2017; Faubert et al., 2017). Using tumor spheroids, Swietach et al. (2009, 2010) demonstrated that in spheroids of up to 300 um in size, CO_2_ released by the mitochondria acts as a major substrate for CAIX activity rather than lactic acid accumulation. The source of CO_2_ can either be from the tumor cells or can be provided to anaerobic regions by surrounding aerobic cells.

Carbonic anhydrase IX establishes a pH gradient of alkaline intracellular pH and acidic extracellular pH in tumor cells that helps in survival and tumor growth (Chiche et al., 2009). Maintenance of intracellular pH by CAIX is critical to support glycolysis and help cancer cells to adapt under hypoxia (Benej et al., 2020). Numerous studies have shown that CAs associate with acid/base transporters to form a temporary complex called transport metabolon (Deitmer and Becker, 2013). CAs form a transport metabolon with MCTs to effectively shuttle the protons from and to MCT, and enhance its activity (Klier et al., 2014). In CAIX, the 18 Glu and 8 Asp residues in the PG domain have been proposed to act as proton antenna or proton collectors (Ames et al., 2018), whereas the His200 in the catalytic domain facilitates the proton shuttle from the catalytic center into surrounding space, and support MCT activity (Jamali et al., 2015). Another important transport metabolon in the context of CAIX is the bicarbonate metabolon that involves the association of CAIX with bicarbonate transporters. CAIX co-localizes and functionally cooperates with the bicarbonate transporter, NbCe1 (SLC4A4), in the invadopodia to achieve an alkaline pH that promotes invadopodia formation (Debreova et al., 2019). Additionally, CAIX interacts with matrix metalloproteinase 14 (MMP14) in invadopodia. MMP14 is a proteolytic enzyme that degrades the extracellular matrix (ECM) and its activation is important for invadopodial function. The association of CAIX with MMP14 provides protons for MMP14 activation and therefore helps in the invadopodial function (Swayampakula et al., 2017). The increased MMP14 activity, coupled with the intracellular alkalinization within the invadopodia, aids in invadopodia elongation and therefore in tumor cell invasion.

The sodium-bicarbonate transporter, NBCn1 (also known as SLC4A7) is a high confidence interactor of CAIX that emerged in the BioID study. Genome-wide association studies have shown NBCn1 to be a causative gene in breast cancer (Ahmed et al., 2009). NBCn1 functions as an acid extruder and creates a favorable pH gradient in tumors (Boedtkjer et al., 2013; Lee et al., 2016). Furthermore, loss of function studies by the genetic depletion of NBCn1 has elucidated its role in tumor growth (Lee et al., 2016) and cell cycle progression (Flinck et al., 2018). Considering the importance of this bicarbonate transporter in regulating pH in tumor cells, it may mediate an important function by forming a bicarbonate metabolon with CAIX. However, to this date, the role of this interaction remains uninvestigated.

## Caix and Amino Acid Transport

**FIGURE 1:**
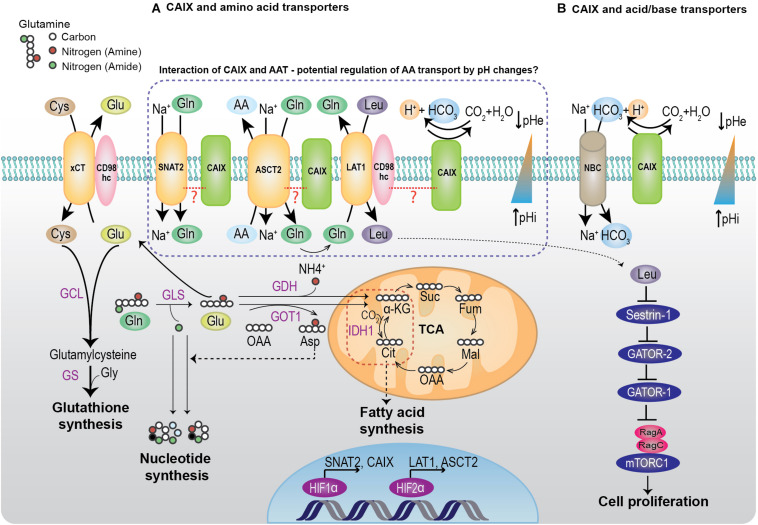
Interaction of CAIX with amino acid transporters and acid/base regulators from BioID. **(A)** Under hypoxia or low amino acid conditions, cells upregulate the expression of SNAT2 and ASCT2 to increase Gln uptake. Normally, Gln can be utilized for biosynthetic reactions such as nucleotide synthesis, bioenergetic reactions by entering the TCA, or for REDOX reactions by glutathione synthesis. Under hypoxia, Gln is utilized for fatty acid synthesis by the reductive carboxylation of α-KG to citrate by IDH1. Alternatively, the intracellular glutamine can be utilized for importing essential amino acids such as leucine, by coupling the transport activity of ASCT2 with LAT1. The imported leucine can bind to a leucine sensor, Sestrin2, removing its inhibitory effect on the RagA/B and activate mTORC1 (Wolfson et al., 2016). The activated mTORC1 promotes protein translation and cell proliferation. **(B)** Hypoxic cells upregulate CAs, MCTs, and acid/base transporters to buffer the intracellular pH changes that occur due to the accumulation of metabolic acids such as CO_2_ and lactic acid (see text). CAIX mediates the reversible conversion of CO_2_ to proton and bicarbonate. This reaction is coupled with the bicarbonate import through NBC, thereby creating a pH gradient of alkaline intracellular pH and acidic extracellular pH that is favorable for cell survival and growth. xCT, cysteine/glutamate transporter; SNAT2, sodium coupled neutral amino acid transporter; ASCT2, alanine serine cysteine transporter 2; LAT1, L-type amino acid transporter; CAIX, carbonic anhydrase IX; NBC, sodium coupled bicarbonate transporter; GLS, glutaminase; GDH, glutamate dehydrogenase; GOT, glutamic oxaloacetic transaminase; GCL, glutamate-cysteine ligase; GS, glutathione synthase; IDH1, Isocitrate dehydrogenase 1.

Hypoxic zones in the tumor have a restricted supply of nutrients and therefore continually adapt to metabolize various nutrients to maintain their biologic functions (Samanta and Semenza, 2018). Amino acids are a major source of carbon and nitrogen for the biosynthesis of various macromolecules (Figure 1). In this section, we will discuss three AATs that were identified as potential interactors of CAIX from the BioID screen (Table 1). We will describe the role and regulation of these transporters in cancer, and then discuss how these transporters may work coordinatively with CAIX in the hypoxic TME.

**TABLE 1:** List of potential CAIX interactors with role in nutrient or metabolite transport function from the BioID.

**Functional class**	**Gene symbol**	**Gene name**	**Biological role**
Amino acid transporters	SLC3A2 or CD98hc	Solute carrier family 3 member 2 or Cluster differentiation 98 heavy chain	Amino acid transport heavy chain subunit. Forms a complex with light chain subunit to create functional heteromeric amino acid transporter
	SLC7A5 or LAT1	Solute carrier family 7 member 5 or L-type amino acid transporter 1	Sodium independent transport of large neutral amino acids such as Leu, Ile, Val, His, Met, Trp, and Phe Tyr (Kanai et al., 1998)
	SLC1A5 or ASCT2	Solute carrier family 1 member 5, ASCT2 or Alanine Serine Cysteine transporter 2	Sodium-dependent transport of neutral amino acids such as Glu, Gln, Ala, Ser, Thr, Val, and Gly (Utsunomiya-Tate et al., 1996)
	SLC38A2 or SNAT2	Solute carrier family 38 member 2 or Sodium coupled neutral amino acid transporter 2	Sodium-dependent transport of neutral amino acids such as Ala, Gln, Ser, Met, Asn, Cys, Gly, His, and Pro (Mackenzie and Erickson, 2004)
Acid/base transporter	SLC4A7 or NBCn1	Solute carrier family 4 member 7 or Sodium bicarbonate cotransporter 3	Sodium bicarbonate cotransport

### ASCT2

The Alanine Serine Cysteine Transporter 2 (ASCT2) aka SLC1A5, is a plasma membrane amino acid transporter that mediates sodium-dependent antiport of neutral amino acids. Despite what the name suggests, ASCT2 preferentially transports glutamine, while cysteine acts as a modulator of the transport (Utsunomiya-Tate et al., 1996; Scalise et al., 2018). ASCT2 is a trimeric protein comprising a scaffold domain that enables the interaction between protomers, and a transport domain that helps in the amino acid transport (Garaeva et al., 2018). As one of the major glutamine transporters in cells, ASCT2 is ubiquitously expressed across various tissues in the body and plays a crucial role in mediating cellular functions such as hematopoietic stem cell differentiation (Oburoglu et al., 2014) and T-cell activation (Nakaya et al., 2014; Poffenberger et al., 2014). Increased expression of ASCT2 is observed in several cancer types and is associated with poor prognosis (Witte et al., 2002; Shimizu et al., 2014; Kaira et al., 2015b; Liu et al., 2015; Sun et al., 2016; Bernhardt et al., 2017). The upregulated cellular expression of ASCT2 in cancer is mediated by oncogenic signals such as Kirsten rat sarcoma (K-Ras) (Toda et al., 2017) and myelocytomatosis (N-Myc) (Ren et al., 2015). K-Ras plays an important role in mediating various growth signaling pathways in cells. Mutation in K-Ras is shown to upregulate the expression of ASCT2 and promote cell proliferation in colorectal cancer (Toda et al., 2017). N-Myc, on the other hand, is a transcription factor that drives the expression of genes involved in cell proliferation. Ren et al. (2015) showed N-Myc to upregulate ASCT2 expression by directly binding to its promoter region. In addition to oncogenic signals, cellular stress such as amino acid starvation can also upregulate ASCT2 expression. Under amino acid deprivation, a stress response transcription factor called activating transcription factor 4 (ATF4) binds to ASCT2 promoter and increases ASCT2 expression (Ren et al., 2015). Functional studies using *in vitro* and *in vivo* models have shown ASCT2 inhibition to effectively reduce tumor growth in various types of cancer (van Geldermalsen et al., 2016; Marshall et al., 2017; Ye et al., 2018) by attenuating the mechanistic Target of Raptor (mTOR) signaling pathway (Figure 1; Wang et al., 2014, 2015). Furthermore, ASCT2 has been shown to facilitate Gln uptake in cancer stem cells and promote tumor growth in pancreatic ductal adenocarcinoma (PDAC) (Wang V.M. et al., 2019). Based on this evidence, it can be concluded that ASCT2 plays an important role in tumorigenesis and is an attractive candidate to target cancer. Over the years, several drug candidates to target ASCT2 have been discovered. However, identifying drugs that selectively target ASCT2 has been a challenge due to limited structural studies until recently (Jiang et al., 2020). The recent development of an antagonist, V9302 by Schulte et al. (2018) has shown promise in targeting ASCT2 (Scopelliti et al., 2018).

### SNAT2

Sodium coupled neutral amino acid transporter 2 (SNAT2) aka SLC38A2, mediates uniport of neutral amino acids including glutamine in a sodium-dependent manner (Mackenzie and Erickson, 2004). It comprises of 11 hydrophobic membrane-spanning domains with an extracellular C-terminus and an intracellular N-terminus (Ge et al., 2018). SNAT2 expression is regulated by extracellular amino acid. Under amino acid deprivation, the global translation is halted, with a concomitant increase in the ATF4 translation. The binding of ATF4 to the amino acid response element (AARE) on the SNAT2 promoter increases SNAT2 expression (Palii et al., 2006). Conversely, SNAT2 can sense the presence of amino acids such as Tyr and Gln, and inhibit its expression (Hyde et al., 2007; Hundal and Taylor, 2009). Besides extracellular amino acid, SNAT2 expression is regulated by endoplasmic reticulum stress (ERS). In breast cancer cells, paclitaxel-induced ERS causes a ubiquitin ligase, RNFα to associate with SNAT2 and ASCT2, and cause their degradation. This leads to decreased Gln uptake, decreased proliferation, and increased cell death in the tumor cells (Jeon et al., 2015; Moses and Neckers, 2015). Studies from Broer’s group have elucidated that SNAT2 expression increases upon the disruption of ASCT2 transporter activity (Broer et al., 2016, 2019), thereby classifying SNAT2 as a rescue transporter. In addition to its role as a Gln transporter, SNAT2 has shown to play an important role in transporting Ala into PDAC cells from the surrounding pancreatic stellate cells (Parker et al., 2020). Furthermore, genetic depletion of SNAT2 in PDAC cells decreases the Ala uptake and reduce tumor growth *in vivo* (Parker et al., 2020). These studies highlight the importance of SNAT2 in cancer, however, the clinical relevance of this transporter in cancers remains unexplored.

### LAT1

The L-type amino acid transporter (LAT1) aka SLC7A5, is a heterodimeric amino acid transporter that mediates a sodium independent antiport of neutral essential amino acids (Kanai et al., 1998; Wagner et al., 2001). It comprises a heavy chain subunit called cluster differentiation 98 (CD98hc) that associates with a light chain subunit such as LAT1. The heavy chain consists of an extracellular C-terminus, a transmembrane helix, and an intracellular NH2 terminus. The light chain, on the other hand, has 12 transmembrane domains with both COOH and the NH2 termini facing the intracellular space (Yan et al., 2019). While LAT1 is expressed across various tissues, it is highly expressed at the blood-brain barrier and functions in amino acid transport to the brain (Boado et al., 1999). The importance of LAT1 in tumor growth was elucidated by Cormerais et al., using knock out models for LAT1 and CD98hc. This study showed the genetic disruption of LAT1 to decrease leucine uptake and inhibit mTORC1, causing decreased tumor growth *in vivo*. N-Myc upregulates LAT1 expression by directly binding to the promoter region of LAT1. The resulting amino acid uptake promotes the sustained translation of Myc, thereby working in a feed-forward loop that helps in tumorigenesis (Yue et al., 2017). LAT1 expression is associated with poor prognosis (Kaira et al., 2015a; Shimizu et al., 2015) and resistance to chemotherapy in solid tumors (Altan et al., 2018). Downregulation of LAT1 has been shown to decrease cell growth (Marshall et al., 2016) invasion, and migration (Janpipatkul et al., 2014). Targeting LAT1 using a small molecule inhibitor, JPH203 has shown success in pre-clinical trials and was recently evaluated in clinical phase trial 1. Although the sample size was low in this clinical trial, the drug was well tolerated and showed promise in targeting LAT1 in patients with advanced solid tumors (Okano et al., 2018).

### Potential Role of CAIX in Amino Acid Transport Regulation

As discussed above, AATs play important roles in promoting tumor progression, and their interaction with CAIX suggests an important mode of functional regulation that requires further investigation.

It is well-known that Gln is an important amino acid in cancer metabolism and several cancer types rely on Gln, which is called Gln addiction (Wise and Thompson, 2010). In hypoxic tumor cells, Gln is channeled for lipid biosynthesis to support cell proliferation (Figure 1). This process is mediated by the reductive carboxylation of α-ketoglutarate (α-KG) to citrate, and the subsequent entry of citrate into lipogenesis (Metallo et al., 2011). Furthermore, it is shown that Gln carbon and nitrogen are efficiently metabolized to support lipid biosynthesis under hypoxia (Wang Y. et al., 2019). The glutamine transporter SNAT2 is upregulated under hypoxia (Morotti et al., 2019) and support glutamine uptake in cancer cells. Interestingly, studies have shown that SNAT2 compensates for the loss of function of ASCT2 (Broer et al., 2019). Moreover, ASCT2 and LAT1 function as obligatory transporters, in which, the influx through one transporter is coupled to the efflux through the second transporter (Nicklin et al., 2009). These data suggest that these three AATs work cooperatively in the TME to promote cancer progression (Figure 1). To our knowledge, there is no existing evidence in the literature on the functional role of CAIX in AA transport, although the identification of potential interactions between CAIX and these AATs suggests that reciprocal functional regulations may be important hallmarks for tumor progression. Considering the pivotal role of CAIX in pH regulation, it is probable that the pH gradient mediated by CAIX in the tumor could influence the function of these AATs. In fact, the transport function of these AATs are influenced by pH, however, the effects are different. The amino acid transport by SNAT2 is sensitive to extracellular pH, where increased extracellular protons compete with sodium ions and impede the SNAT2 activity. This pH sensitivity of SNAT2 is shown to be mediated by the presence of His residues at the C-terminus (Baird et al., 2006). In contrast, glutamine transport by ASCT2 is not hugely influenced by pH (Utsunomiya-Tate et al., 1996). However, ASCT2 also mediates Glu antiport, and this is highly pH-dependent (Utsunomiya-Tate et al., 1996). At a pH gradient of low extracellular pH (6.0) and high intracellular pH (7.0), the Glu influx increased in proteoliposomes containing ASCT2 (Scalise et al., 2020). In addition to altering the transport by ASCT2, changes in pH has shown to impact the expression of ASCT2. Under chronic acidosis, ASCT2 is upregulated by HIF2α and cause a shift in the cancer cell metabolism to favor reductive glutamine metabolism (Corbet et al., 2014). These studies suggest that the pH gradient across the tumor could influence the function of AATs, however, whether CAIX’s pH regulatory role influences the coordinative interplay of these AATs remains a topic of future investigation. Investigating the effect of loss of function of CAIX on amino acid transport and metabolism could reveal the importance of CAIX’s interaction with the AATs. Such studies could be of importance in highly aggressive cancers like pancreatic cancer that have complex metabolic network (Sperb et al., 2020) and are difficult to treat. CAIX expression (Strapcova et al., 2020) and its function in altering tumoral pH (Cruz-Monserrate et al., 2014) are shown to be important in the early events of pancreatic carcinogenesis. Furthermore, ASCT2 and SNAT2 play an important role in importing AA in pancreatic cancer, as described earlier in this section. Therefore, it is plausible that CAIX and AATs coordinate their functions to support tumor metabolism and promote pancreatic cancer progression.

One of the interesting findings of the BioID CAIX interactome (Swayampakula et al., 2017) was the potential interaction of LAT1 and CD98hc with CAIX. Modulation of LAT1 function and transport of neutral amino acids by CAIX, perhaps in the context of complexes with integrins, which also associate with CD98hc (Fenczik et al., 2001; Feral et al., 2005), may have significant effects on cellular growth through the regulation of protein translation by mTORC1. While this possibility needs to be investigated in further detail, it is interesting that inhibition of CAIX modulates mTORC1 signaling in breast cancer cells grown in 3D cultures (Lock et al., 2013).

## Conclusion

Since the seminal findings of Otto Warburg on altered metabolism in cancer, the concept of metabolic reprogramming in cancer has evolved and led to a better understanding of the complex nature of cancer metabolism (Martinez-Outschoorn et al., 2017). Research on the role of metabolite transport has progressed tremendously and unraveled the importance of numerous nutrient and acid/base transporters in the TME (Ganapathy et al., 2009; Bhutia et al., 2015; Becker and Deitmer, 2020). Understanding the interaction of these metabolic proteins in the TME would be beneficial in identifying novel targets for effective therapy. Based on our interactome study, we identified the potential interaction of nutrient transporters and acid/base transporters with CAIX in the hypoxic TME. CAIX is an important pH regulatory protein in the TME that mediates tumor progression in several solid tumors. The CAIX/CAXII specific small-molecule inhibitor, SLC-0111 (Pacchiano et al., 2011; Supuran, 2018) has shown promising effect on suppressing tumor growth and metastasis by itself and in combination with conventional chemotherapeutic drugs (Boyd et al., 2017; McDonald et al., 2019) or immune checkpoint inhibitors (Chafe et al., 2019). Currently, SLC-0111 has completed the Phase-I clinical trial and progressed into a Phase-Ib trial (ClinicalTrials.gov Identifier: NCT03450018) in combination with gemcitabine in CAIX-positive pancreatic cancer patients (McDonald et al., 2020).Furthermore, recent studies have shown that the metabolic plasticity in solid tumors offers adaptation and resistance to single therapy strategies by initiating compensatory mechanisms, however, this is effectively overcome by combinatorial therapy (Biancur et al., 2017; Momcilovic et al., 2018). Therefore, investigating the interaction of CAIX with these nutrient transporters might open new avenues of co-targeting strategies for the treatment of solid tumors.

## Author Contributions

GV and SD conceived and designed the manuscript and revised and approved the final manuscript. GV drafted the manuscript. Both authors contributed to the article and approved the submitted version.

## Conflict of Interest

The authors declare that the research was conducted in the absence of any commercial or financial relationships that could be construed as a potential conflict of interest.

## References

[B1] Ahmed S. Thomas G. Ghoussaini M. Healey C. S. Humphreys M. K. Platte R. (2009). Newly discovered breast cancer susceptibility loci on 3p24 and 17q23.2. *Nat. Genet.* 41 585–590. 10.1038/ng.354 19330027 PMC2748125

[B2] Altan B. Kaira K. Watanabe A. Kubo N. Bao P. Dolgormaa G. (2018). Relationship between LAT1 expression and resistance to chemotherapy in pancreatic ductal adenocarcinoma. *Cancer Chemother. Pharmacol.* 81 141–153. 10.1007/s00280-017-3477-4 29149426

[B3] Alterio V. Di Fiore A. D’Ambrosio K. Supuran C. T. De Simone G. (2012). Multiple binding modes of inhibitors to carbonic anhydrases: how to design specific drugs targeting 15 different isoforms? *Chem. Rev.* 112 4421–4468. 10.1021/cr200176r 22607219

[B4] Alterio V. Hilvo M. Di Fiore A. Supuran C. T. Pan P. Parkkila S. (2009). Crystal structure of the catalytic domain of the tumor-associated human carbonic anhydrase IX. *Proc. Natl. Acad. Sci.* 106:16233. 10.1073/pnas.0908301106 19805286 PMC2752527

[B5] Ames S. Pastorekova S. Becker H. M. (2018). The proteoglycan-like domain of carbonic anhydrase IX mediates non-catalytic facilitation of lactate transport in cancer cells. *Oncotarget* 9 27940–27957. 10.18632/oncotarget.25371 29963253 PMC6021347

[B6] Baird F. E. Pinilla-Tenas J. J. Ogilvie W. L. J. Ganapathy V. Hundal H. S. Taylor P. M. (2006). Evidence for allosteric regulation of pH-sensitive System A (SNAT2) and System N (SNAT5) amino acid transporter activity involving a conserved histidine residue. *Biochem. J.* 397 369–375. 10.1042/BJ20060026 16629640 PMC1513278

[B7] Becker H. M. Deitmer J. W. (2020). Transport Metabolons and Acid/Base Balance in Tumor Cells. *Cancers* 12:899. 10.3390/cancers12040899 32272695 PMC7226098

[B8] Benej M. Svastova E. Banova R. Kopacek J. Gibadulinova A. Kery M. (2020). CA IX Stabilizes Intracellular pH to Maintain Metabolic Reprogramming and Proliferation in Hypoxia. *Front. Oncol.* 10:1462. 10.3389/fonc.2020.01462 32983978 PMC7493625

[B9] Bernhardt S. Bayerlova M. Vetter M. Wachter A. Mitra D. Hanf V. (2017). Proteomic profiling of breast cancer metabolism identifies SHMT2 and ASCT2 as prognostic factors. *Breast Cancer Res.* 19:112. 10.1186/s13058-017-0905-7 29020998 PMC5637318

[B10] Bhutia Y. D. Babu E. Ramachandran S. Ganapathy V. (2015). Amino Acid transporters in cancer and their relevance to “glutamine addiction”: novel targets for the design of a new class of anticancer drugs. *Cancer Res.* 75 1782–1788. 10.1158/0008-5472.Can-14-3745 25855379

[B11] Biancur D. E. Paulo J. A. Malachowska B. Quiles Del, Rey M. Sousa C. M. (2017). Compensatory metabolic networks in pancreatic cancers upon perturbation of glutamine metabolism. *Nat. Commun.* 8:15965. 10.1038/ncomms15965 28671190 PMC5500878

[B12] Boado R. J. Li J. Y. Nagaya M. Zhang C. Pardridge W. M. (1999). Selective expression of the large neutral amino acid transporter at the blood-brain barrier. *Proc. Natl. Acad. Sci. U S A* 96 12079–12084. 10.1073/pnas.96.21.12079 10518579 PMC18415

[B13] Boedtkjer E. Moreira J. M. Mele M. Vahl P. Wielenga V. T. Christiansen P. M. (2013). Contribution of Na+,HCO3(-)-cotransport to cellular pH control in human breast cancer: a role for the breast cancer susceptibility locus NBCn1 (SLC4A7). *Int. J. Cancer* 132 1288–1299. 10.1002/ijc.27782 22907202

[B14] Boyd N. H. Walker K. Fried J. Hackney J. R. McDonald P. C. Benavides G. A. (2017). Addition of carbonic anhydrase 9 inhibitor SLC-0111 to temozolomide treatment delays glioblastoma growth in vivo. *JCI Insight* 2:e92928. 10.1172/jci.insight.92928 29263302 PMC5752277

[B15] Briggs K. J. Koivunen P. Cao S. Backus K. M. Olenchock B. A. Patel H. (2016). Paracrine Induction of HIF by Glutamate in Breast Cancer: EglN1 Senses Cysteine. *Cell* 166 126–139. 10.1016/j.cell.2016.05.042 27368101 PMC4930557

[B16] Broer A. Gauthier-Coles G. Rahimi F. van Geldermalsen M. Dorsch D. Wegener A. (2019). Ablation of the ASCT2 (SLC1A5) gene encoding a neutral amino acid transporter reveals transporter plasticity and redundancy in cancer cells. *J. Biol. Chem.* 294 4012–4026. 10.1074/jbc.RA118.006378 30635397 PMC6422075

[B17] Broer A. Rahimi F. Broer S. (2016). Deletion of Amino Acid Transporter ASCT2 (SLC1A5) Reveals an Essential Role for Transporters SNAT1 (SLC38A1) and SNAT2 (SLC38A2) to Sustain Glutaminolysis in Cancer Cells. *J. Biol. Chem.* 291 13194–13205. 10.1074/jbc.M115.700534 27129276 PMC4933233

[B18] Chafe S. C. Lou Y. Sceneay J. Vallejo M. Hamilton M. J. McDonald P. C. (2015). Carbonic anhydrase IX promotes myeloid-derived suppressor cell mobilization and establishment of a metastatic niche by stimulating G-CSF production. *Cancer Res.* 75 996–1008. 10.1158/0008-5472.can-14-3000 25623234

[B19] Chafe S. C. McDonald P. C. Saberi S. Nemirovsky O. Venkateswaran G. Burugu S. (2019). Targeting Hypoxia-Induced Carbonic Anhydrase IX Enhances Immune-Checkpoint Blockade Locally and Systemically. *Cancer Immunol. Res.* 7 1064–1078. 10.1158/2326-6066.Cir-18-0657 31088846

[B20] Chia S. K. Wykoff C. C. Watson P. H. Han C. Leek R. D. Pastorek J. (2001). Prognostic significance of a novel hypoxia-regulated marker, carbonic anhydrase IX, in invasive breast carcinoma. *J. Clin. Oncol.* 19 3660–3668. 10.1200/jco.2001.19.16.3660 11504747

[B21] Chiche J. Ilc K. Laferriere J. Trottier E. Dayan F. Mazure N. M. (2009). Hypoxia-inducible carbonic anhydrase IX and XII promote tumor cell growth by counteracting acidosis through the regulation of the intracellular pH. *Cancer Res.* 69 358–368. 10.1158/0008-5472.Can-08-2470 19118021

[B22] Corbet C. Draoui N. Polet F. Pinto A. Drozak X. Riant O. (2014). The SIRT1/HIF2alpha axis drives reductive glutamine metabolism under chronic acidosis and alters tumor response to therapy. *Cancer Res.* 74 5507–5519. 10.1158/0008-5472.can-14-0705 25085245

[B23] Corbet C. Feron O. (2017). Tumour acidosis: from the passenger to the driver’s seat. *Nat. Rev. Cancer* 17 577–593. 10.1038/nrc.2017.77 28912578

[B24] Cruz-Monserrate Z. Roland C. L. Deng D. Arumugam T. Moshnikova A. Andreev O. A. (2014). Targeting pancreatic ductal adenocarcinoma acidic microenvironment. *Sci. Rep.* 4:4410 10.1038/srep04410 PMC3958716 24642931

[B25] Debreova M. Csaderova L. Burikova M. Lukacikova L. Kajanova I. Sedlakova O. (2019). CAIX Regulates Invadopodia Formation through Both a pH-Dependent Mechanism and Interplay with Actin Regulatory Proteins. *Int. J. Mol. Sci.* 20:2745 10.3390/ijms20112745 31167468 PMC6600150

[B26] Deitmer J. Becker H. (2013). Transport metabolons with carbonic anhydrases. *Front. Physiol.* 4:291 10.3389/fphys.2013.00291 PMC3794380 24133456

[B27] DeNicola G. M. Cantley L. C. (2015). Cancer’s Fuel Choice: New Flavors for a Picky Eater. *Mol. Cell* 60 514–523. 10.1016/j.molcel.2015.10.018 26590711 PMC4676726

[B28] Faubert B. Li K. Y. Cai L. Hensley C. T. Kim J. Zacharias L. G. (2017). Lactate Metabolism in Human Lung Tumors. *Cell* 171:358.e–371.e. 10.1016/j.cell.2017.09.019 28985563 PMC5684706

[B29] Fenczik C. A. Zent R. Dellos M. Calderwood D. A. Satriano J. Kelly C. (2001). Distinct domains of CD98hc regulate integrins and amino acid transport. *J. Biol. Chem.* 276 8746–8752. 10.1074/jbc.M011239200 11121428

[B30] Feral C. C. Nishiya N. Fenczik C. A. Stuhlmann H. Slepak M. Ginsberg M. H. (2005). CD98hc (SLC3A2) mediates integrin signaling. *Proc. Natl. Acad. Sci. U S A* 102 355–360. 10.1073/pnas.0404852102 15625115 PMC544283

[B31] Flinck M. Kramer S. H. Schnipper J. Andersen A. P. Pedersen S. F. (2018). The acid-base transport proteins NHE1 and NBCn1 regulate cell cycle progression in human breast cancer cells. *Cell Cycle* 17 1056–1067. 10.1080/15384101.2018.1464850 29895196 PMC6110587

[B32] Ganapathy V. Thangaraju M. Prasad P. D. (2009). Nutrient transporters in cancer: relevance to Warburg hypothesis and beyond. *Pharmacol. Ther.* 121 29–40. 10.1016/j.pharmthera.2008.09.005 18992769

[B33] Garaeva A. A. Oostergetel G. T. Gati C. Guskov A. Paulino C. Slotboom D. J. (2018). Cryo-EM structure of the human neutral amino acid transporter ASCT2. *Nat. Struct. Mol. Biol.* 25 515–521. 10.1038/s41594-018-0076-y 29872227

[B34] Ge Y. Gu Y. Wang J. Zhang Z. (2018). Membrane topology of rat sodium-coupled neutral amino acid transporter 2 (SNAT2). *Biochim. Biophy. Acta Biomembr.* 1860 1460–1469. 10.1016/j.bbamem.2018.04.005 29678469

[B35] Generali D. Fox S. B. Berruti A. Brizzi M. P. Campo L. Bonardi S. (2006). Role of carbonic anhydrase IX expression in prediction of the efficacy and outcome of primary epirubicin/tamoxifen therapy for breast cancer. *Endocr. Relat. Cancer* 13 921–930. 10.1677/erc.1.01216 16954440

[B36] Gibadulinova A. Bullova P. Strnad H. Pohlodek K. Jurkovicova D. Takacova M. (2020). CAIX-Mediated Control of LIN28/let-7 Axis Contributes to Metabolic Adaptation of Breast Cancer Cells to Hypoxia. *Int. J. Mol. Sci.* 21:4299. 10.3390/ijms21124299 32560271 PMC7352761

[B37] Gieling R. G. Babur M. Mamnani L. Burrows N. Telfer B. A. Carta F. (2012). Antimetastatic effect of sulfamate carbonic anhydrase IX inhibitors in breast carcinoma xenografts. *J. Med. Chem.* 55 5591–5600. 10.1021/jm300529u 22621623

[B38] Hundal H. S. Taylor P. M. (2009). Amino acid transceptors: gate keepers of nutrient exchange and regulators of nutrient signaling. *Am. J. Physiol. Endocrinol. Metab.* 296 E603–E613. 10.1152/ajpendo.91002.2008 19158318 PMC2670634

[B39] Hyde R. Cwiklinski E. L. MacAulay K. Taylor P. M. Hundal H. S. (2007). Distinct sensor pathways in the hierarchical control of SNAT2, a putative amino acid transceptor, by amino acid availability. *J. Biol. Chem.* 282 19788–19798. 10.1074/jbc.M611520200 17488712

[B40] Ilie M. Mazure N. M. Hofman V. Ammadi R. E. Ortholan C. Bonnetaud C. (2010). High levels of carbonic anhydrase IX in tumour tissue and plasma are biomarkers of poor prognostic in patients with non-small cell lung cancer. *Br. J. Cancer* 102 1627–1635. 10.1038/sj.bjc.6605690 20461082 PMC2883156

[B41] Jamali S. Klier M. Ames S. Felipe Barros L. McKenna R. Deitmer J. W. (2015). Hypoxia-induced carbonic anhydrase IX facilitates lactate flux in human breast cancer cells by non-catalytic function. *Sci. Rep.* 5:13605. 10.1038/srep13605 26337752 PMC4559800

[B42] Janpipatkul K. Suksen K. Borwornpinyo S. Jearawiriyapaisarn N. Hongeng S. Piyachaturawat P. (2014). Downregulation of LAT1 expression suppresses cholangiocarcinoma cell invasion and migration. *Cell Signal* 26 1668–1679. 10.1016/j.cellsig.2014.04.002 24726839

[B43] Jeon Y. J. Khelifa S. Ratnikov B. Scott D. A. Feng Y. Parisi F. (2015). Regulation of glutamine carrier proteins by RNF5 determines breast cancer response to ER stress-inducing chemotherapies. *Cancer Cell* 27 354–369. 10.1016/j.ccell.2015.02.006 25759021 PMC4356903

[B44] Jiang H. Zhang N. Tang T. Feng F. Sun H. Qu W. (2020). Target the human Alanine/Serine/Cysteine Transporter 2(ASCT2): Achievement and Future for Novel Cancer Therapy. *Pharmacol. Res.* 158:104844. 10.1016/j.phrs.2020.104844 32438035

[B45] Kaira K. Nakamura K. Hirakawa T. Imai H. Tominaga H. Oriuchi N. (2015a). Prognostic significance of L-type amino acid transporter 1 (LAT1) expression in patients with ovarian tumors. *Am. J. Transl. Res.* 7 1161–1171. 26279759 PMC4532748

[B46] Kaira K. Sunose Y. Arakawa K. Sunaga N. Shimizu K. Tominaga H. (2015b). Clinicopathological significance of ASC amino acid transporter-2 expression in pancreatic ductal carcinoma. *Histopathology* 66 234–243. 10.1111/his.12464 24845232

[B47] Kanai Y. Segawa H. Miyamoto K. Uchino H. Takeda E. Endou H. (1998). Expression cloning and characterization of a transporter for large neutral amino acids activated by the heavy chain of 4F2 antigen (CD98). *J. Biol. Chem.* 273 23629–23632. 10.1074/jbc.273.37.23629 9726963

[B48] Klatte T. Seligson D. B. Rao J. Y. Yu H. de Martino M. Kawaoka K. (2009). Carbonic anhydrase IX in bladder cancer: a diagnostic, prognostic, and therapeutic molecular marker. *Cancer* 115 1448–1458. 10.1002/cncr.24163 19195047

[B49] Klier M. Andes F. T. Deitmer J. W. Becker H. M. (2014). Intracellular and extracellular carbonic anhydrases cooperate non-enzymatically to enhance activity of monocarboxylate transporters. *J. Biol. Chem.* 289 2765–2775. 10.1074/jbc.M113.537043 24338019 PMC3908409

[B50] Korkeila E. Talvinen K. Jaakkola P. M. Minn H. Syrjanen K. Sundstrom J. (2009). Expression of carbonic anhydrase IX suggests poor outcome in rectal cancer. *Br. J. Cancer* 100 874–880. 10.1038/sj.bjc.6604949 19240720 PMC2661792

[B51] Koukourakis M. I. Giatromanolaki A. Sivridis E. Simopoulos K. Pastorek J. Wykoff C. C. (2001). Hypoxia-regulated carbonic anhydrase-9 (CA9) relates to poor vascularization and resistance of squamous cell head and neck cancer to chemoradiotherapy. *Clin Cancer Res.* 7 3399–3403. 11705854

[B52] Lee S. Axelsen T. V. Andersen A. P. Vahl P. Pedersen S. F. Boedtkjer E. (2016). Disrupting Na(+), HCO(3)(-)-cotransporter NBCn1 (Slc4a7) delays murine breast cancer development. *Oncogene* 35 2112–2122. 10.1038/onc.2015.273 26212013

[B53] Liu Y. Yang L. An H. Chang Y. Zhang W. Zhu Y. (2015). High expression of Solute Carrier Family 1, member 5 (SLC1A5) is associated with poor prognosis in clear-cell renal cell carcinoma. *Sci. Rep.* 5:16954. 10.1038/srep16954 26599282 PMC4657035

[B54] Lock F. E. McDonald P. C. Lou Y. Serrano I. Chafe S. C. Ostlund C. (2013). Targeting carbonic anhydrase IX depletes breast cancer stem cells within the hypoxic niche. *Oncogene* 32 5210–5219. 10.1038/onc.2012.550 23208505

[B55] Loncaster J. A. Harris A. L. Davidson S. E. Logue J. P. Hunter R. D. Wycoff C. C. (2001). Carbonic anhydrase (CA IX) expression, a potential new intrinsic marker of hypoxia: correlations with tumor oxygen measurements and prognosis in locally advanced carcinoma of the cervix. *Cancer Res.* 61 6394–6399. 11522632

[B56] Lou Y. McDonald P. C. Oloumi A. Chia S. Ostlund C. Ahmadi A. (2011). Targeting tumor hypoxia: suppression of breast tumor growth and metastasis by novel carbonic anhydrase IX inhibitors. *Cancer Res.* 71 3364–3376. 10.1158/0008-5472.can-10-4261 21415165

[B57] Mackenzie B. Erickson J. D. (2004). Sodium-coupled neutral amino acid (System N/A) transporters of the SLC38 gene family. *Pflugers Arch.* 447 784–795. 10.1007/s00424-003-1117-9 12845534

[B58] Marshall A. D. van Geldermalsen M. Otte N. J. Anderson L. A. Lum T. Vellozzi M. A. (2016). LAT1 is a putative therapeutic target in endometrioid endometrial carcinoma. *Int. J. Cancer* 139 2529–2539. 10.1002/ijc.30371 27486861

[B59] Marshall A. D. van Geldermalsen M. Otte N. J. Lum T. Vellozzi M. Thoeng A. (2017). ASCT2 regulates glutamine uptake and cell growth in endometrial carcinoma. *Oncogenesis* 6:e367. 10.1038/oncsis.2017.70 28759021 PMC5541720

[B60] Martinez-Outschoorn U. E. Peiris-Pages M. Pestell R. G. Sotgia F. Lisanti M. P. (2017). Cancer metabolism: a therapeutic perspective. *Nat. Rev. Clin. Oncol.* 14 11–31. 10.1038/nrclinonc.2016.60 27141887

[B61] Mboge M. Y. Mahon B. P. McKenna R. Frost S. C. (2018). Carbonic Anhydrases: Role in pH Control and Cancer. *Metabolites* 8:19 10.3390/metabo8010019 29495652 PMC5876008

[B62] McDonald P. C. Chafe S. C. Brown W. S. Saberi S. Swayampakula M. Venkateswaran G. (2019). Regulation of pH by Carbonic Anhydrase 9 Mediates Survival of Pancreatic Cancer Cells With Activated KRAS in Response to Hypoxia. *Gastroenterology* 157 823–837 10.1053/j.gastro.2019.05.004 31078621

[B63] McDonald P. C. Chia S. Bedard P. L. Chu Q. Lyle M. Tang L. (2020). A Phase 1 Study of SLC-0111, a Novel Inhibitor of Carbonic Anhydrase IX, in Patients With Advanced Solid Tumors. *Am. J. Clin. Oncol.* 43 484–490. 10.1097/coc.0000000000000691 32251122 PMC7323835

[B64] McDonald P. C. Dedhar S. (2014). Carbonic anhydrase IX (CAIX) as a mediator of hypoxia-induced stress response in cancer cells. *Subcell. Biochem.* 75 255–269. 10.1007/978-94-007-7359-2_13 24146383

[B65] McDonald P. C. Winum J. Y. Supuran C. T. Dedhar S. (2012). Recent developments in targeting carbonic anhydrase IX for cancer therapeutics. *Oncotarget* 3 84–97. 10.18632/oncotarget.422 22289741 PMC3292895

[B66] McIntyre A. Patiar S. Wigfield S. Li J. L. Ledaki I. Turley H. (2012). Carbonic anhydrase IX promotes tumor growth and necrosis in vivo and inhibition enhances anti-VEGF therapy. *Clin. Cancer Res.* 18 3100–3111. 10.1158/1078-0432.Ccr-11-1877 22498007 PMC3367109

[B67] Metallo C. M. Gameiro P. A. Bell E. L. Mattaini K. R. Yang J. Hiller K. (2011). Reductive glutamine metabolism by IDH1 mediates lipogenesis under hypoxia. *Nature* 481 380–384. 10.1038/nature10602 22101433 PMC3710581

[B68] Michiels C. Tellier C. Feron O. (2016). Cycling hypoxia: A key feature of the tumor microenvironment. *Biochim. Biophys. Acta* 1866 76–86. 10.1016/j.bbcan.2016.06.004 27343712

[B69] Momcilovic M. Bailey S. T. Lee J. T. Fishbein M. C. Braas D. Go J. (2018). The GSK3 Signaling Axis Regulates Adaptive Glutamine Metabolism in Lung Squamous Cell Carcinoma. *Cancer Cell* 33:905.e–921.e. 10.1016/j.ccell.2018.04.002 29763624 PMC6451645

[B70] Morotti M. Bridges E. Valli A. Choudhry H. Sheldon H. Wigfield S. (2019). Hypoxia-induced switch in SNAT2/SLC38A2 regulation generates endocrine resistance in breast cancer. *Proc. Natl. Acad. Sci. U S A* 116 12452–12461. 10.1073/pnas.1818521116 31152137 PMC6589752

[B71] Moses M. A. Neckers L. (2015). The GLU that holds cancer together: targeting GLUtamine transporters in breast cancer. *Cancer Cell* 27 317–319. 10.1016/j.ccell.2015.02.010 25759015

[B72] Nakaya M. Xiao Y. Zhou X. Chang J.-H. Chang M. Cheng X. (2014). Inflammatory T Cell Responses Rely on Amino Acid Transporter ASCT2 Facilitation of Glutamine Uptake and mTORC1 Kinase Activation. *Immunity* 40 692–705. 10.1016/j.immuni.2014.04.007 24792914 PMC4074507

[B73] Nakazawa M. S. Keith B. Simon M. C. (2016). Oxygen availability and metabolic adaptations. *Nat. Rev. Cancer* 16 663–673. 10.1038/nrc.2016.84 27658636 PMC5546320

[B74] Neri D. Supuran C. T. (2011). Interfering with pH regulation in tumours as a therapeutic strategy. *Nat. Rev. Drug Discov.* 10 767–777. 10.1038/nrd3554 21921921

[B75] Nicklin P. Bergman P. Zhang B. Triantafellow E. Wang H. Nyfeler B. (2009). Bidirectional transport of amino acids regulates mTOR and autophagy. *Cell* 136 521–534. 10.1016/j.cell.2008.11.044 19203585 PMC3733119

[B76] Oburoglu L. Tardito S. Fritz V. de Barros S. C. Merida P. Craveiro M. (2014). Glucose and glutamine metabolism regulate human hematopoietic stem cell lineage specification. *Cell Stem Cell* 15 169–184. 10.1016/j.stem.2014.06.002 24953180

[B77] Okano N. Kawai K. Yamauchi Y. Kobayashi T. Naruge D. Nagashima F. (2018). First-in-human phase I study of JPH203 in patients with advanced solid tumors. *Journal of Clinical Oncology* 36 419–419. 10.1200/JCO.2018.36.4_suppl.419

[B78] Opavský R. Pastoreková S. Zelnìk V. R. Gibadulinová A. Stanbridge E. J. Závada J. (1996). HumanMN/CA9Gene, a Novel Member of the Carbonic Anhydrase Family: Structure and Exon to Protein Domain Relationships. *Genomics* 33 480–487. 10.1006/geno.1996.0223 8661007

[B79] Pacchiano F. Carta F. McDonald P. C. Lou Y. Vullo D. Scozzafava A. (2011). Ureido-substituted benzenesulfonamides potently inhibit carbonic anhydrase IX and show antimetastatic activity in a model of breast cancer metastasis. *J. Med. Chem.* 54 1896–1902. 10.1021/jm101541x 21361354

[B80] Palii S. S. Thiaville M. M. Pan Y. X. Zhong C. Kilberg M. S. (2006). Characterization of the amino acid response element within the human sodium-coupled neutral amino acid transporter 2 (SNAT2) System A transporter gene. *Biochem. J.* 395 517–527. 10.1042/bj20051867 16445384 PMC1462688

[B81] Panisova E. Kery M. Sedlakova O. Brisson L. Debreova M. Sboarina M. (2017). Lactate stimulates CA IX expression in normoxic cancer cells. *Oncotarget* 8 77819–77835. 10.18632/oncotarget.20836 29100428 PMC5652817

[B82] Parker S. J. Amendola C. R. Hollinshead K. E. R. Yu Q. Yamamoto K. Encarnación-Rosado J. (2020). Selective Alanine Transporter Utilization Creates a Targetable Metabolic Niche in Pancreatic Cancer. *Cancer Discov.* 10 1018–1037. 10.1158/2159-8290.Cd-19-0959 32341021 PMC7334074

[B83] Peppicelli S. Andreucci E. Ruzzolini J. Bianchini F. Nediani C. Supuran C. T. (2020). The Carbonic Anhydrase IX inhibitor SLC-0111 as emerging agent against the mesenchymal stem cell-derived pro-survival effects on melanoma cells. *J. Enzyme Inhib. Med. Chem.* 35 1185–1193. 10.1080/14756366.2020.1764549 32396749 PMC7269050

[B84] Poffenberger Maya C. Jones Russell G. (2014). Amino Acids Fuel T Cell-Mediated Inflammation. *Immunity* 40 635–637. 10.1016/j.immuni.2014.04.017 24837098

[B85] Ren P. Yue M. Xiao D. Xiu R. Gan L. Liu H. (2015). ATF4 and N-Myc coordinate glutamine metabolism in MYCN-amplified neuroblastoma cells through ASCT2 activation. *J. Pathol.* 235 90–100. 10.1002/path.4429 25142020

[B86] Roux K. J. Kim D. I. Raida M. Burke B. (2012). A promiscuous biotin ligase fusion protein identifies proximal and interacting proteins in mammalian cells. *J. Cell Biol.* 196 801–810. 10.1083/jcb.201112098 22412018 PMC3308701

[B87] Samanta D. Semenza G. L. (2018). Metabolic adaptation of cancer and immune cells mediated by hypoxia-inducible factors. *Biochim. Biophy. Acta Rev. Cancer* 1870 15–22. 10.1016/j.bbcan.2018.07.002 30006019

[B88] Scalise M. Mazza T. Pappacoda G. Pochini L. Cosco J. Rovella F. (2020). The Human SLC1A5 Neutral Amino Acid Transporter Catalyzes a pH-Dependent Glutamate/Glutamine Antiport, as Well. *Front. Cell Dev. Biol.* 8:603 10.3389/fcell.2020.00603 32733894 PMC7360689

[B89] Scalise M. Pochini L. Console L. Losso M. A. Indiveri C. (2018). The Human SLC1A5 (ASCT2) Amino Acid Transporter: From Function to Structure and Role in Cell Biology. *Front. Cell Dev. Biol.* 6 96–96. 10.3389/fcell.2018.00096 30234109 PMC6131531

[B90] Schulte M. L. Fu A. Zhao P. Li J. Geng L. Smith S. T. (2018). Pharmacological blockade of ASCT2-dependent glutamine transport leads to antitumor efficacy in preclinical models. *Nat. Med.* 24 194–202. 10.1038/nm.4464 29334372 PMC5803339

[B91] Scopelliti A. J. Font J. Vandenberg R. J. Boudker O. Ryan R. M. (2018). Structural characterisation reveals insights into substrate recognition by the glutamine transporter ASCT2/SLC1A5. *Nat. Commun.* 9:38. 10.1038/s41467-017-02444-w 29295993 PMC5750217

[B92] Shimizu A. Kaira K. Kato M. Yasuda M. Takahashi A. Tominaga H. (2015). Prognostic significance of L-type amino acid transporter 1 (LAT1) expression in cutaneous melanoma. *Melanoma Res.* 25 399–405. 10.1097/cmr.0000000000000181 26237765

[B93] Shimizu K. Kaira K. Tomizawa Y. Sunaga N. Kawashima O. Oriuchi N. (2014). ASC amino-acid transporter 2 (ASCT2) as a novel prognostic marker in non-small cell lung cancer. *Br. J. Cancer* 110 2030–2039. 10.1038/bjc.2014.88 24603303 PMC3992511

[B94] Sperb N. Tsesmelis M. Wirth T. (2020). Crosstalk between Tumor and Stromal Cells in Pancreatic Ductal Adenocarcinoma. *Int. J. Mol. Sci.* 21:5486 10.3390/ijms21155486 32752017 PMC7432853

[B95] Strapcova S. Takacova M. Csaderova L. Martinelli P. Lukacikova L. Gal V. (2020). Clinical and Pre-Clinical Evidence of Carbonic Anhydrase IX in Pancreatic Cancer and Its High Expression in Pre-Cancerous Lesions. *Cancers* 12:2005 10.3390/cancers12082005 32707920 PMC7464147

[B96] Sun H. W. Yu X. J. Wu W. C. Chen J. Shi M. Zheng L. (2016). GLUT1 and ASCT2 as Predictors for Prognosis of Hepatocellular Carcinoma. *PLoS One* 11:e0168907. 10.1371/journal.pone.0168907 28036362 PMC5201247

[B97] Sun X. Wang M. Wang M. Yao L. Li X. Dong H. (2020). Role of Proton-Coupled Monocarboxylate Transporters in Cancer: From Metabolic Crosstalk to Therapeutic Potential. *Front. Cell Dev. Biol.* 8:651 10.3389/fcell.2020.00651 32766253 PMC7379837

[B98] Supuran C. T. (2018). Carbonic anhydrase inhibitors as emerging agents for the treatment and imaging of hypoxic tumors. *Expert Opin. Investig. Drugs* 27 963–970. 10.1080/13543784.2018.1548608 30426805

[B99] Swayampakula M. McDonald P. C. Vallejo M. Coyaud E. Chafe S. C. Westerback A. (2017). The interactome of metabolic enzyme carbonic anhydrase IX reveals novel roles in tumor cell migration and invadopodia/MMP14-mediated invasion. *Oncogene* 36 6244–6261. 10.1038/onc.2017.219 28692057 PMC5684442

[B100] Swietach P. Hulikova A. Vaughan-Jones R. D. Harris A. L. (2010). New insights into the physiological role of carbonic anhydrase IX in tumour pH regulation. *Oncogene* 29 6509–6521. 10.1038/onc.2010.455 20890298

[B101] Swietach P. Patiar S. Supuran C. T. Harris A. L. Vaughan-Jones R. D. (2009). The role of carbonic anhydrase 9 in regulating extracellular and intracellular ph in three-dimensional tumor cell growths. *J. Biol. Chem.* 284 20299–20310. 10.1074/jbc.M109.006478 19458084 PMC2740455

[B102] Tan E. Y. Yan M. Campo L. Han C. Takano E. Turley H. (2009). The key hypoxia regulated gene CAIX is upregulated in basal-like breast tumours and is associated with resistance to chemotherapy. *Br. J. Cancer* 100 405–411. 10.1038/sj.bjc.6604844 19165203 PMC2634728

[B103] Toda K. Nishikawa G. Iwamoto M. Itatani Y. Takahashi R. Sakai Y. (2017). Clinical Role of ASCT2 (SLC1A5) in KRAS-Mutated Colorectal Cancer. *Int. J. Mol. Sci.* 18:1632. 10.3390/ijms18081632 28749408 PMC5578022

[B104] Utsunomiya-Tate N. Endou H. Kanai Y. (1996). Cloning and functional characterization of a system ASC-like Na+-dependent neutral amino acid transporter. *J. Biol. Chem.* 271 14883–14890. 8662767 10.1074/jbc.271.25.14883

[B105] van Geldermalsen M. Wang Q. Nagarajah R. Marshall A. D. Thoeng A. Gao D. (2016). ASCT2/SLC1A5 controls glutamine uptake and tumour growth in triple-negative basal-like breast cancer. *Oncogene* 35 3201–3208. 10.1038/onc.2015.381 26455325 PMC4914826

[B106] Wagner C. A. Lang F. Broer S. (2001). Function and structure of heterodimeric amino acid transporters. *Am. J. Physiol. Cell Physiol.* 281 C1077–C1093. 11546643 10.1152/ajpcell.2001.281.4.C1077

[B107] Wang Q. Beaumont K. A. Otte N. J. Font J. Bailey C. G. van Geldermalsen M. (2014). Targeting glutamine transport to suppress melanoma cell growth. *Int. J. Cancer* 135 1060–1071. 10.1002/ijc.28749 24531984

[B108] Wang Q. Hardie R. A. Hoy A. J. van Geldermalsen M. Gao D. Fazli L. (2015). Targeting ASCT2-mediated glutamine uptake blocks prostate cancer growth and tumour development. *J. Pathol.* 236 278–289. 10.1002/path.4518 25693838 PMC4973854

[B109] Wang V. M. Ferreira R. M. M. Almagro J. Evan T. Legrave N. Zaw Thin M. (2019). CD9 identifies pancreatic cancer stem cells and modulates glutamine metabolism to fuel tumour growth. *Nat. Cell Biol.* 21 1425–1435. 10.1038/s41556-019-0407-1 31685994 PMC6944508

[B110] Wang Y. Bai C. Ruan Y. Liu M. Chu Q. Qiu L. (2019). Coordinative metabolism of glutamine carbon and nitrogen in proliferating cancer cells under hypoxia. *Nat. Commun.* 10:201. 10.1038/s41467-018-08033-9 30643150 PMC6331631

[B111] Ward P. S. Thompson C. B. (2012). Metabolic reprogramming: a cancer hallmark even warburg did not anticipate. *Cancer Cell* 21 297–308. 10.1016/j.ccr.2012.02.014 22439925 PMC3311998

[B112] Wise D. R. Thompson C. B. (2010). Glutamine addiction: a new therapeutic target in cancer. *Trends Biochem. Sci.* 35 427–433. 10.1016/j.tibs.2010.05.003 20570523 PMC2917518

[B113] Witte D. Ali N. Carlson N. Younes M. (2002). Overexpression of the neutral amino acid transporter ASCT2 in human colorectal adenocarcinoma. *Anticancer Res.* 22 2555–2557. 12529963

[B114] Wolfson R. L. Chantranupong L. Saxton R. A. Shen K. Scaria S. M. Cantor J. R. (2016). Sestrin2 is a leucine sensor for the mTORC1 pathway. *Science* 351 43–48. 10.1126/science.aab2674 26449471 PMC4698017

[B115] Wykoff C. C. Beasley N. J. Watson P. H. Turner K. J. Pastorek J. Sibtain A. (2000). Hypoxia-inducible expression of tumor-associated carbonic anhydrases. *Cancer Res.* 60 7075–7083. 11156414

[B116] Xie H. Simon M. C. (2017). Oxygen availability and metabolic reprogramming in cancer. *J. Biol. Chem.* 292 16825–16832. 10.1074/jbc.R117.799973 28842498 PMC5641881

[B117] Yan R. Zhao X. Lei J. Zhou Q. (2019). Structure of the human LAT1-4F2hc heteromeric amino acid transporter complex. *Nature* 568 127–130. 10.1038/s41586-019-1011-z 30867591

[B118] Ye J. Huang Q. Xu J. Huang J. Wang J. Zhong W. (2018). Targeting of glutamine transporter ASCT2 and glutamine synthetase suppresses gastric cancer cell growth. *J. Cancer Res. Clin. Oncol.* 144 821–833. 10.1007/s00432-018-2605-9 29435734 PMC5916984

[B119] Yue M. Jiang J. Gao P. Liu H. Qing G. (2017). Oncogenic MYC Activates a Feedforward Regulatory Loop Promoting Essential Amino Acid Metabolism and Tumorigenesis. *Cell Rep.* 21 3819–3832. 10.1016/j.celrep.2017.12.002 29281830

